# How parenting styles shape adolescents’ test anxiety: the mediating role of academic grit

**DOI:** 10.3389/fpsyg.2026.1750833

**Published:** 2026-03-26

**Authors:** Sheng Zhang, Yue Zhao, Junqing Wen, Ziming Wang, Tao Xin

**Affiliations:** 1 Collaborative Innovation Center of Assessment for Basic Education Quality, Beijing Normal University, Beijing, China; 2 Haidian Institute of Education Sciences, Beijing, China

**Keywords:** academic grit, parenting styles, partial correlation network analysis, structural equation modeling, test anxiety

## Abstract

This study examines the associations among parenting styles, academic grit, and adolescent test anxiety. Drawing on a sample of 26,897 seventh-graders from 90 Beijing junior secondary schools, the research utilized self-report measures of parental care, parental control, academic grit, and test anxiety. Adopting an integrative methodological perspective, the study combined partial correlation network analysis to examine the structural configuration of the psychological system and structural equation modeling (SEM) to estimate theoretically specified indirect associations. Network analysis indicated that academic grit-particularly the perseverance of effort dimension-occupied the most structurally central position within the associative network, linking parenting variables and test anxiety. Parental care and perseverance showed strong positive connections, whereas perseverance and consistency of interest were negatively connected with test anxiety. Within the SEM framework, parental care was positively associated with both dimensions of grit, which in turn were negatively associated with test anxiety. Parental control showed negative associations with grit and positive indirect associations with anxiety. Perseverance demonstrated stronger indirect associations with test anxiety than consistency of interest. Together, these findings suggest that academic grit is embedded in the relational structure connecting family context and test-related emotional experiences. By integrating network modeling with structural equation analysis, this study delineates both the structural prominence of grit within an associative system and the indirect relational pathways linking parenting styles and test anxiety.

## 1 Introduction

In the field of psychology, family education is regarded as one of the most fundamental and enduring socialization forces shaping individual psychological development (Bronfenbrenner, 1979a,b; Härkönen, 2001). Scholars have long been concerned with these variables, particularly parenting style, which is defined as the overall emotional climate of family functioning and child-rearing (Maccoby, 1994). It encompasses two fundamental dimensions: support and control (Baumrind, 1971).

Previous studies have indicated that parenting styles influence students’ non-cognitive qualities, which in turn affect their academic achievement (Wei et al., 2025; Howard et al., 2019; Martín Quintana et al., 2023). However, beyond academic performance, emotional adjustment in evaluative contexts warrants greater scholarly attention. Test anxiety has emerged as a widespread psychological concern among Chinese adolescents. As early as 2011, national survey data indicated that approximately 13% of Chinese students experienced clinically significant levels of test anxiety (Shen et al., 2011). More recent evidence from the SSES 2019 survey suggests that this proportion has risen substantially, with over 30% of students reporting notable test-related anxiety (He and Qing, 2024). This upward trend underscores that test anxiety is no longer a marginal phenomenon but a prevalent psychological issue affecting students’ well-being and holistic development.

Within East Asian cultural contexts characterized by high academic expectations and competitive examination systems, test anxiety represents a particularly salient dimension of students’ emotional functioning (Stankov, 2010). Yet, despite its growing prevalence, most existing research has focused primarily on academic achievement outcomes, leaving the emotional consequences of family socialization comparatively underexplored. To further understand the impact of family education on students’ learning states, it is important to note that while many studies support the mediating role of academic grit in the relationship between parenting styles and student outcomes, some have reported non-significant pathways-for instance, among Taiwanese samples (Lin and Chang, 2017). Moreover, few large-scale studies have systematically examined whether academic grit explains how parenting styles relate specifically to test anxiety.

### 1.1 Academic grit and test anxiety

The concept of grit was first introduced by Duckworth et al. (2007), defined as a combination of sustained passion and persistent effort toward long-term goals. Within this framework, passion describes an individual’s long-term dedication to a specific goal that can span months or even years, and perseverance describes the ability to sustain effort and continue pursuing that goal in the face of failure and setbacks. Theoretically, grit encompasses a sense of confidence in one’s ability to overcome challenges such as examinations. In contrast, test anxiety has been found to correlate with lower self-confidence and an external locus of control (Hembree, 1988).

Subsequent research has extended the original domain-general conceptualization of grit to more context-specific forms, including academic grit or learning grit, which emphasize sustained engagement and perseverance within educational settings (Clark and Malecki, 2019; Datu et al., 2016). Academic grit retains the core dual components of perseverance and long-term goal commitment but situates them explicitly within the learning domain. Empirical evidence suggests substantial conceptual overlap between general grit and academic grit, with the latter representing a contextualized manifestation of the broader construct. Thus, academic grit can be understood not as a distinct theoretical entity but as a domain-specific operationalization of grit in school-based achievement contexts.

In the localization of grit research, scholars have pointed out that individuals in East Asian cultures may interpret “perseverance” and “responsibility” differently from the interest-driven conception prevalent in Western contexts. Consequently, both the measurement and underlying mechanisms of grit should be examined in light of specific cultural backgrounds (Ma et al., 2020). Moreover, following the implementation of the Chinese “Double Reduction” policy and ongoing reforms in educational assessment, the sources of test anxiety have expanded beyond exam difficulty to encompass broader social and familial pressures. As a non-cognitive trait closely related to self-control, grit emphasizes sustained motivation toward long-term goals (Duckworth et al., 2014). Compared with other psychological constructs such as growth mindset or self-efficacy, grit reflects a form of psychological endurance that sustains individuals’ engagement in the face of challenges and setbacks (Duckworth et al., 2007; Credé et al., 2017). Its buffering effect on test anxiety highlights the distinctive value of non-cognitive qualities in educational adaptation. Within evaluative contexts, grit is regarded as a critical psychological resource for resisting anxiety. On one hand, grit enhances goal orientation and cognitive resource integration, thereby reducing threat perception during tests; on the other hand, it strengthens emotional regulation, enabling students to maintain psychological resilience under academic pressure (Disabato et al., 2019; Tang et al., 2019).

In particular, the perseverance of effort dimension has been found to correlate strongly with emotional stability, self-expectation, and test confidence, significantly predicting lower levels of anxiety (Datu et al., 2016; Luthans et al., 2019). In contrast, the consistency of interests dimension-reflecting sustained identification with long-term goals and learning values-requires further empirical clarification regarding its buffering role against test anxiety, as its predictive power tends to be weaker or non-significant in collectivist cultural contexts (Datu et al., 2016; Credé et al., 2017). Test anxiety may be understood as a breakdown of regulatory control under evaluative threat, and perseverance, as sustained goal-directed regulation in the face of difficulty, represents a theoretically relevant mechanism linking parenting environments to adolescents’ anxiety responses in testing situations.

### 1.2 Parenting style and test anxiety

Test anxiety differs from general anxiety in that it emphasizes the situational and contextual nature of its occurrence—namely, evaluative and testing environments. In such highly goal-oriented contexts, pressure often arises not only from within the individual but also from external sources. A study conducted in the United Kingdom revealed that a portion of students’ anxiety is transmitted through instructional interactions (Connor, 2003), indicating that test anxiety is not merely a learning-related emotional issue but also reflects deeper interconnections among personality, family, and social factors.

Parenting style is defined as the overall emotional climate of family functioning and child-rearing. Following the dimensional tradition of parenting research, it can be conceptualized along two fundamental dimensions: parental care and parental control. Parental care refers to warmth, emotional support, and responsiveness toward the child, whereas parental control reflects the degree of monitoring, regulation, and behavioral demands imposed by parents. These dimensions correspond broadly to the classical constructs of responsiveness and demandingness proposed by Baumrind (1971), but are operationalized in the present study as care and control to maintain consistency with the measurement and analytical framework used in this research. Based on these two dimensions, Baumrind proposed three primary parenting styles—authoritative, authoritarian, and permissive. From a psychodynamic standpoint, parenting constitutes one of the most critical early socialization experiences that shape several key psychological mechanisms (Blatt and Homann, 1992). Authoritative parents, through clear rules and warm support, help children develop a stable ego and superego, fostering a balanced sense of reality and morality. In contrast, authoritarian parenting may lead to the formation of an overly harsh superego, resulting in guilt, repression, and even compulsive personality traits. Permissive or neglectful parenting, on the other hand, may weaken ego functions, leading to impulsivity, emotional dysregulation, and poor reality testing.

In this study, we adopt a dimensional approach to parenting, operationalized through the two continuous dimensions of parental care and parental control. This approach is adopted for several reasons. First, treating parenting along continuous dimensions allows for more precise modeling of individual differences and interactions with adolescent outcomes. Second, continuous measures provide greater sensitivity to subtle variations in parenting behavior, which is particularly useful when linking parenting to cognitive and emotional processes such as attention regulation and test anxiety. Finally, from a practical perspective, dimensional indicators are more actionable in educational and family interventions: whereas broad typological categories are relatively abstract, continuous dimensions can directly guide specific parenting strategies (e.g., increasing warmth or reducing controlling behaviors) to mitigate adolescents’ test anxiety.

Bronfenbrenner’s Ecological Systems Theory conceptualizes parenting as embedded within nested environmental systems. Adolescents’ emotional adjustment is shaped by immediate family interactions (microsystem) as well as school contexts, educational policies, and broader sociocultural values (macrosystem) (Bronfenbrenner, 1979a,b; Smetana, 2017). In examination-oriented societies, strong cultural emphases on achievement intensify parental expectations and may increase adolescents’ vulnerability to test anxiety.

Furthermore, Bandura’s Social Learning Theory highlights modeling and reinforcement mechanisms through which parenting influences anxiety development. Children may internalize parents’ attitudes toward achievement, stress, and failure through observational learning. When parents display excessive worry about academic outcomes or equate performance with personal worth, adolescents may adopt similar evaluative schemas and maladaptive coping patterns.

Empirical research by Lamborn et al. (1991) also demonstrated that parenting styles are significantly associated with various developmental outcomes in adolescence, including academic achievement, psychosocial adjustment, internalizing distress, and behavioral problems. More recent studies further suggest that parenting practices may not only directly affect students’ academic stress and anxiety levels (Pomerantz et al., 2007), but also exert indirect effects on their psychological adjustment through mediating variables such as learning motivation, self-efficacy, or Grit (Yang et al., 2025; Tang et al., 2022; Shengyao et al., 2024).

### 1.3 Parenting style and academic grit

In recent years, increasing attention has been devoted to the foundational role of family parenting in the development of adolescents’ non-cognitive traits. Parenting styles not only influence students’ emotional regulation and behavioral expressions but also profoundly shape their intrinsic motivation and personality dispositions (Wei and Liu, 2022). Empirical studies have shown that the authoritative parenting style—characterized by high levels of both control and warmth—facilitates the cultivation of perseverance and serves as an important family basis for the development of Grit (Duckworth et al., 2014). Specifically, parental emotional responsiveness, encouragement of exploration, and moderate autonomy support have been found to be closely associated with adolescents’ high performance in both the perseverance of effort and consistency of interests.

Self-Determination Theory (SDT) offers a robust framework for explaining how parenting influences the development of perseverance. According to SDT, the satisfaction of three basic psychological needs-autonomy, competence, and relatedness-supports sustained intrinsic motivation (Ryan and Deci, 2000). Authoritative parenting, through warmth and autonomy support, fosters these needs and strengthens adolescents’ internalization of long-term goals. In contrast, highly controlling parenting undermines autonomy and weakens intrinsic motivation, thereby reducing sustained engagement in challenging tasks (Grolnick and Ryan, 1989; Baumrind, 1999). When adolescents’ psychological needs are thwarted, goal commitment becomes externally regulated and fragile, which conflicts with the enduring effort and goal stability central to grit.

Particularly in environments that neglect individuals’ needs for autonomy, adolescents tend to exhibit lower goal commitment and weaker resilience, standing in sharp contrast to the long-term engagement and goal consistency required by grit. These findings suggest that parents’ everyday socialization practices not only shape students’ immediate learning behaviors but also, through their influence on personality traits such as grit, determine adolescents’ deeper psychological endurance and developmental trajectories when facing academic challenges.

### 1.4 Current study

The present study employs partial correlation network analysis to explore whether grit functions as a core hub within the mediating pathways linking family factors and emotional states. Unlike traditional structural modeling approaches, network analysis allows for the examination of the overall relational structure among variables, revealing both direct and indirect associations and identifying key bridge nodes that play central roles within the system. Through this perspective, the current study aims to provide a more nuanced depiction of grit’s unique role in the parenting–anxiety pathway.

Taken together, grit may not only serve as a mediating variable between parenting styles and test anxiety but may also exert a significant direct predictive effect on students’ test anxiety. Furthermore, the structural centrality of grit within the variable network has not yet been systematically validated, particularly among large samples of Chinese adolescents. To address these gaps, the following hypotheses are proposed:

*H1*: Academic grit is negatively associated with students’ test anxiety.

Previous research has consistently demonstrated that parenting styles significantly predict the development of academic grit, and that academic grit itself is closely associated with test anxiety. These empirical patterns raise the question of whether academic grit functions as an intermediary construct linking parenting styles and anxiety-related outcomes.

Among the various theoretical perspectives discussed earlier, Self-Determination Theory provides a particularly relevant framework for understanding the motivational processes underlying academic grit. According to Self-Determination Theory (Ryan and Deci, 2000), parenting practices are closely related to adolescents’ motivational internalization processes. Parental care is associated with greater satisfaction of basic psychological needs and higher levels of autonomous engagement, whereas parental control is more often linked to externally regulated forms of motivation. These motivational orientations are relevant to the development of sustained academic engagement and long-term goal pursuit. In evaluative contexts, perseverance of effort reflects sustained goal-directed regulation when students encounter academic difficulty. It has been negatively associated with the cognitive and emotional components of test anxiety. This pattern suggests that perseverance of effort may represent one pathway through which parenting styles are indirectly related to anxiety-related outcomes. Consistency of interests reflects stability in long-term goal orientation and value commitment. Although its functional implications may vary across cultural contexts, stable academic identification has been associated with lower goal ambivalence and reduced evaluative distress. This dimension may therefore constitute a second potential intermediary construct linking parenting styles and test anxiety.

Taken together, these theoretical considerations provide a rationale for examining the mediating roles of perseverance of effort and consistency of interests in the association between parenting styles and test anxiety. Accordingly, the following hypotheses are proposed:

*H2a*: Perseverance of Effort mediates the relationship between Parenting Styles and test anxiety.

*H2b*: Consistency of Interests mediates the relationship between Parenting Styles and test anxiety.

## 2 Methods

### 2.1 Data and sample

This study adopted a cross-sectional survey design based on large-scale online questionnaire data. A cluster sampling strategy was employed to select 90 junior secondary schools (including branch campuses) across Haidian District, Beijing—one of the most educationally representative urban areas in China and a core region reflecting East Asian examination-oriented culture. A total of 27,650 seventh-grade students participated in the survey. After data cleaning and the removal of 753 invalid responses, 26,897 valid cases were retained, resulting in an effective response rate of 98.22%. A total of 13,712 male participants (50.98%) and 13,185 female participants (49.02%) were included in the study.

The survey was administered through a secure online platform with the assistance of trained school staff and class teachers. Data were collected in September 2024. Participation was voluntary and anonymous. Written informed consent for participation in this study was obtained from the participants’ legal guardians or next of kin prior to data collection. Ethical review and approval was not required for the study on human participants in accordance with the local legislation and institutional requirements. All data collection procedures adhered to institutional ethical standards, ensuring confidentiality and minimal risk to participants.

With respect to inclusion and exclusion criteria, all enrolled seventh-grade students in the selected schools were eligible to participate. No additional inclusion or exclusion criteria (e.g., academic performance thresholds, health status, or family background restrictions) were applied, as the study aimed to capture a population-based cohort within the district. Exclusion was limited to responses deemed invalid based on predefined data-quality standards.

### 2.2 Measures and variables

#### 2.2.1 Academic grit

Academic grit was assessed using a revised version of the Short Grit Scale (Grit-S) developed by Duckworth and Quinn (2009). The adapted Chinese version consisted of six items covering two subdimensions: perseverance of effort and consistency of interest. All items were rated on a five-point Likert scale ranging from 1 (“not at all like me”) to 5 (“very much like me”). Higher scores indicated higher levels of academic grit. Previous validation studies have confirmed the scale’s reliability and cultural applicability among Chinese adolescents (Jiang et al., 2023). In the present study, Cronbach’s *α* values were 0.804 for perseverance of effort and 0.734 for consistency of interest, demonstrating good internal consistency.

#### 2.2.2 Parenting style

Parenting style was measured using an adapted version of Steinberg’s Parenting Style Scale (Steinberg et al., 1994), including two subscales: parental care and parental control. The parental care subscale (4 items) assessed students’ perceived warmth, support, and emotional responsiveness from their parents (e.g., “My parents talk to me in a gentle and friendly tone”). The parental control subscale (4 items) assessed perceived parental monitoring and restriction (e.g., “My parents don’t want me to grow up”). Responses were given on a five-point Likert scale (1 = “strongly disagree,” 5 = “strongly agree”). Cronbach’s *α* values for the care and control dimensions were 0.909 and 0.860, respectively.

#### 2.2.3 Test anxiety

Test anxiety was assessed using a simplified version of the Multidimensional test anxiety Scale (MTAS; Putwain et al., 2021). The adapted measure contained three representative items capturing cognitive worry, emotional tension, and physiological arousal (e.g., “During exams, I feel nervous if I cannot recall what I have learned”). Items were rated on a five-point Likert scale from 1 (“not at all true”) to 5 (“very true”). Higher scores reflected higher levels of test anxiety. The scale demonstrated good reliability in this study (Cronbach’s *α* = 0.858).

### 2.3 Analytic strategy

#### 2.3.1 Descriptive

Descriptive analyses were first performed to examine the basic distributional characteristics of all study variables, including parental care, parental control, perseverance of effort, consistency of interests, and test anxiety. Prior to conducting the main analyses, we assessed the normality, range, and variability of the variables, as well as potential issues with missing values or univariate outliers. Internal consistency reliability for each scale was also evaluated. These descriptive procedures ensured that the data met the assumptions required for subsequent network modeling and structural equation analysis. Detailed descriptive results are reported in the Results section.

#### 2.3.2 Network analysis

Network analysis was conducted to examine the structural associations among parenting styles, academic grit, and test anxiety. All analyses were performed in R using the qgraph and bootnet packages. Because the variables were measured on ordinal Likert-type scales, a polychoric correlation matrix was computed as the basis for network estimation.

A Gaussian graphical model (GGM) was estimated using the Extended Bayesian Information Criterion graphical LASSO (EBICglasso) procedure. This regularization approach shrinks trivially small edges toward zero, producing a sparse and interpretable network that minimizes the risk of spurious associations. The tuning parameter (*γ*) was set to the conventional value of 0.5 to balance goodness-of-fit and parsimony.

To evaluate the structural importance of variables within the network, two weighted centrality indices were computed: the Onnela coefficient and the Zhang coefficient. The Onnela coefficient quantifies the topological contribution of each node by emphasizing the relative strength of edges in local connectivity patterns, providing a measure of how strongly a node is integrated within its immediate neighborhood (Onnela et al., 2005). In contrast, the Zhang coefficient captures global connectivity by assessing the overall weighted sum of associations linked to each node while discounting spurious weak connections (Zhang and Horvath, 2005). Together, these indices offer complementary perspectives on local and global prominence within the psychological network and are commonly used in weighted undirected networks to evaluate centrality robustness.

Network stability and accuracy were evaluated via nonparametric bootstrapping procedures. Edge-weight precision was inspected using 95% confidence intervals, and centrality stability was assessed using the correlation stability (CS) coefficient. These procedures ensured the robustness and interpretability of the network parameters. All settings followed recommended guidelines for psychological network analysis.

#### 2.3.3 SEM

Structural equation modeling (SEM) was conducted in Python to examine the proposed relationships among parenting styles, academic grit, and test anxiety. The analyses were implemented using the semopy package, which supports latent variable estimation under maximum likelihood and provides standardized path coefficients, indirect effects, and model fit indices aligned with those produced by established SEM software.

In the model, parental care and parental control were treated as exogenous variables; perseverance of effort and consistency of interest were included as mediators; and test anxiety was specified as the outcome. Each latent construct was defined by its observed indicators, and all factor loadings were freely estimated. Residuals were assumed to be independent unless theoretical considerations indicated the need for covariance.

Because the items were ordinal, polychoric correlation matrices were computed using the pingouin package and used as the input for model estimation. Parameters were estimated with robust maximum likelihood (MLR), which adjusts standard errors for non-normality. Model fit was evaluated using commonly reported indices—the comparative fit index (CFI), Tucker–Lewis index (TLI), root-mean-square error of approximation (RMSEA), and standardized root-mean-square residual (SRMR)—following widely accepted criteria for determining adequate fit.

Indirect effects were tested using 5,000 bootstrap resamples with semopy’s built-in bootstrap function, and bias-corrected confidence intervals were calculated to ensure accurate mediation estimates. Standardized coefficients were reported for all structural paths to support clearer comparisons across variables. All analytic procedures followed current recommendations for conducting mediation analysis with Python-based SEM tools.

Finally, gender was included as a control variable in the model to account for its potential influence on test anxiety. This adjustment ensured that the estimated associations between parenting, grit, and anxiety reflected effects above and beyond gender-related differences.

## 3 Results

The results are organized into three sections. First, the reliability and validity analyses for all measurement scales are reported to ensure the robustness of the instruments. Second, descriptive statistics and intercorrelations among the key variables—parenting styles, academic grit, and test anxiety—are presented. Third, the results of the partial correlation network analysis and the structural equation modeling (SEM) are reported in detail, providing both the structural relationships among variables and the verification of the mediating effects of academic grit.

### 3.1 Descriptive statistics

Descriptive statistics and Pearson correlations among the main study variables are presented in Table 1. Overall, students reported a moderate level of academic grit and a relatively low to moderate level of test anxiety. Parental care was positively associated with both dimensions of grit, whereas parental control showed a negative association. Consistent with theoretical expectations, academic grit was negatively correlated with test anxiety, indicating that students with higher perseverance and more stable interest tended to experience less anxiety in evaluative situations.

**Table 1:** Descriptive statistics and partial correlation result (*N* = 26,897).

Variable	*M*	SD	Test anxiety	Parental control	Parental care	Academic performance	Perseverance of effort	Consistency of interests
Test anxiety	3.31	1.19	0	−0.016	−0.125	0	−0.178	−0.153
Parental control	2.23	1.13	−0.016	0	−0.115	−0.074	−0.020	−0.217
Parental care	3.99	1.03	−0.125	−0.115	0	0.019	0.338	0.017
Academic performance	222.1	40.03	0	−0.074	0.019	0	0.19	0.078
Perseverance of effort	3.99	0.88	−0.178	−0.020	0.338	0.19	0	0.057
Consistency of interests	3.19	1.02	−0.153	−0.217	0.017	0.078	0.057	0

### 3.2 Partial correlation network analysis

A partial correlation network was constructed to examine the conditional associations among Parenting Styles, academic grit (including its two subdimensions), Academic Performance and test anxiety after controlling for shared variance across variables. The network consisted of six nodes and 14 non-zero edges out of 15 possible connections (sparsity = 0.067), indicating a highly interconnected structure.

The edge weight matrix is presented in Table 1. Negative values indicate inverse partial correlations, whereas positive values represent positive conditional associations. Test anxiety was negatively associated with perseverance of effort (−0.178) and consistency of interests (−0.153), suggesting that both dimensions of academic grit function as protective factors against anxiety in evaluative contexts. Test anxiety also showed a negative association with parental care (−0.125), whereas its association with parental control was negligible (−0.016).

Among the parenting variables, parental care demonstrated the strongest positive association with perseverance of effort (0.338), representing the largest edge in the network. This finding indicates that warm and supportive parenting is closely linked to adolescents’ sustained effort. In contrast, parental control showed a negative association with consistency of interests (−0.217), suggesting that controlling practices may undermine stable long-term interest commitment.

The overall Academic Performance was positively connected to perseverance (0.190) and consistency (0.078).

Centrality indices are presented in Table 2. Perseverance of effort exhibited the highest values across all centrality metrics, including betweenness (1.581), closeness (1.462), strength (1.683), and expected influence (1.255). This pattern indicates that perseverance occupies the most structurally central position in the network. In topological terms, it functions as the primary bridge connecting parenting variables (parental care and parental control) with adolescents’ psychological and academic outcomes (test anxiety and academic performance). Its high expected influence further suggests that its connections are predominantly positive and integrative within the system.

**Table 2:** Node centrality indices in the network.

Variable	Betweenness	Closeness	Strength	Expected influence
Academic performance	−1.581	−0.979	−1.155	0.775
Perseverance of effort	1.581	1.462	1.683	1.255
Parental care	0	0.662	0.55	0.553
Parental control	0	−0.808	−0.603	−1.039
Test anxiety	0	0.447	−0.401	−1.122
Consistency of interests	0	−0.784	−0.073	−0.422

In contrast, consistency of interests demonstrated relatively low strength (−0.073) and expected influence (−0.422), indicating a more peripheral and weakly connected position. Although it remains negatively associated with test anxiety, its structural contribution to overall network connectivity appears limited.

Test anxiety showed negative strength (−0.401) and expected influence (−1.122), consistent with its role as an outcome-oriented node characterized primarily by negative conditional associations. Its low betweenness indicates that it does not serve as a bridging construct but is instead positioned downstream in the network structure.

Regarding parenting variables, parental care demonstrated moderate centrality (strength = 0.550; expected influence = 0.553), reflecting its positive and relatively distributed connections within the network-particularly with perseverance. By contrast, parental control displayed negative strength (−0.603) and expected influence (−1.039), indicating that its connections are predominantly adverse in direction and less structurally integrative.

Academic performance showed relatively low closeness (−0.979) and strength (−1.155), suggesting that it occupies a more peripheral position in the network and does not function as a structural hub. This pattern is consistent with its conceptual role as an outcome variable rather than a bridging mechanism.

Classification metrics based on Barrat, Onnela, WS, and Zhang coefficients are reported in Table 3. Test anxiety exhibited the highest Onnela (1.576) and Zhang (1.705) coefficients, indicating strong local clustering among its immediate neighbors. This suggests that the nodes directly connected to test anxiety are also interconnected, forming a relatively cohesive local structure.

**Table 3:** Node classification metrics.

Variable	Barrat	Onnela	WS	Zhang
Academic performance	1.1	−0.091	1.291	0.288
Perseverance of effort	−1.198	0.643	−0.645	−0.774
Parental care	−0.045	−0.193	−0.645	0.371
Parental control	0.106	−1.271	−0.645	−0.729
Test anxiety	1.1	1.576	1.291	1.705
Consistency of interests	−1.064	−0.665	−0.645	−0.861

In contrast, perseverance of effort showed comparatively lower clustering coefficients but high betweenness centrality, reflecting a more integrative bridging profile rather than dense local clustering. This pattern reinforces its role as a connector across different conceptual domains (parenting and outcomes) rather than as a node embedded within a tightly knit local cluster.

Parenting variables demonstrated differentiated clustering profiles, with parental control showing lower Onnela values and more negative weighted clustering indicators, consistent with its less cohesive and predominantly negative relational pattern.

Taken together, the network results indicate that perseverance of effort occupies the most structurally central position within the Parenting style-academic grit-test anxiety framework. Among all nodes, perseverance demonstrated the strongest integrative and bridging properties, suggesting that it represents the primary structural linkage through which parenting factors are connected to adolescents’ emotional and academic outcomes. Although both grit dimensions were negatively associated with test anxiety, perseverance showed substantially greater connectivity and influence within the overall network configuration. Its particularly strong connection with parental care further underscores its role as a key relational mechanism embedded within the family context.

Consistency of interests emerged as comparatively peripheral, with weaker overall connectivity and limited bridging capacity. This pattern suggests that, while it remains relevant to the system, it contributes less to the structural integration of parenting and outcome variables. Academic performance, although linked to perseverance, did not occupy a central topological position, which aligns with its conceptual role as an outcome indicator rather than a core psychological mechanism within the network.

### 3.3 Structural equation modeling (SEM) results

A structural equation model (SEM) was employed to examine the mediating role of the two dimensions of academic grit—perseverance of effort and consistency of interest—in the relationships between parenting styles and test anxiety. Maximum likelihood (ML) estimation was used, and standardized coefficients are reported below. The SEM model demonstrated an overall acceptable level of fit (CFI = 0.952, RMSEA = 0.086, SRMR = 0.027). Although the RMSEA exceeded the conventional cutoff of 0.08, such elevations are common in large-sample SEMs with few indicators per latent construct—particularly the three-item test anxiety measure and the use of ordinal polychoric input matrices. The CFI exceeded the criterion for good fit (0.95), and the SRMR indicated excellent fit (0.027). Although the RMSEA was slightly elevated, such a pattern is common in large-sample models with brief scales, especially when using polychoric correlations and robust maximum likelihood estimation. In contrast, the CFI and SRMR met standards for good fit, indicating that the model satisfactorily captured the latent structure. Overall, the fit indices support the robustness of the hypothesized pathways (Table 4).

**Table 4:** Model fit indices.

Estimate	CFI	RMSEA	SRMR
Estimated	0.952	0.086	0.027

#### 3.3.1 Direct effects

**Figure 1:**
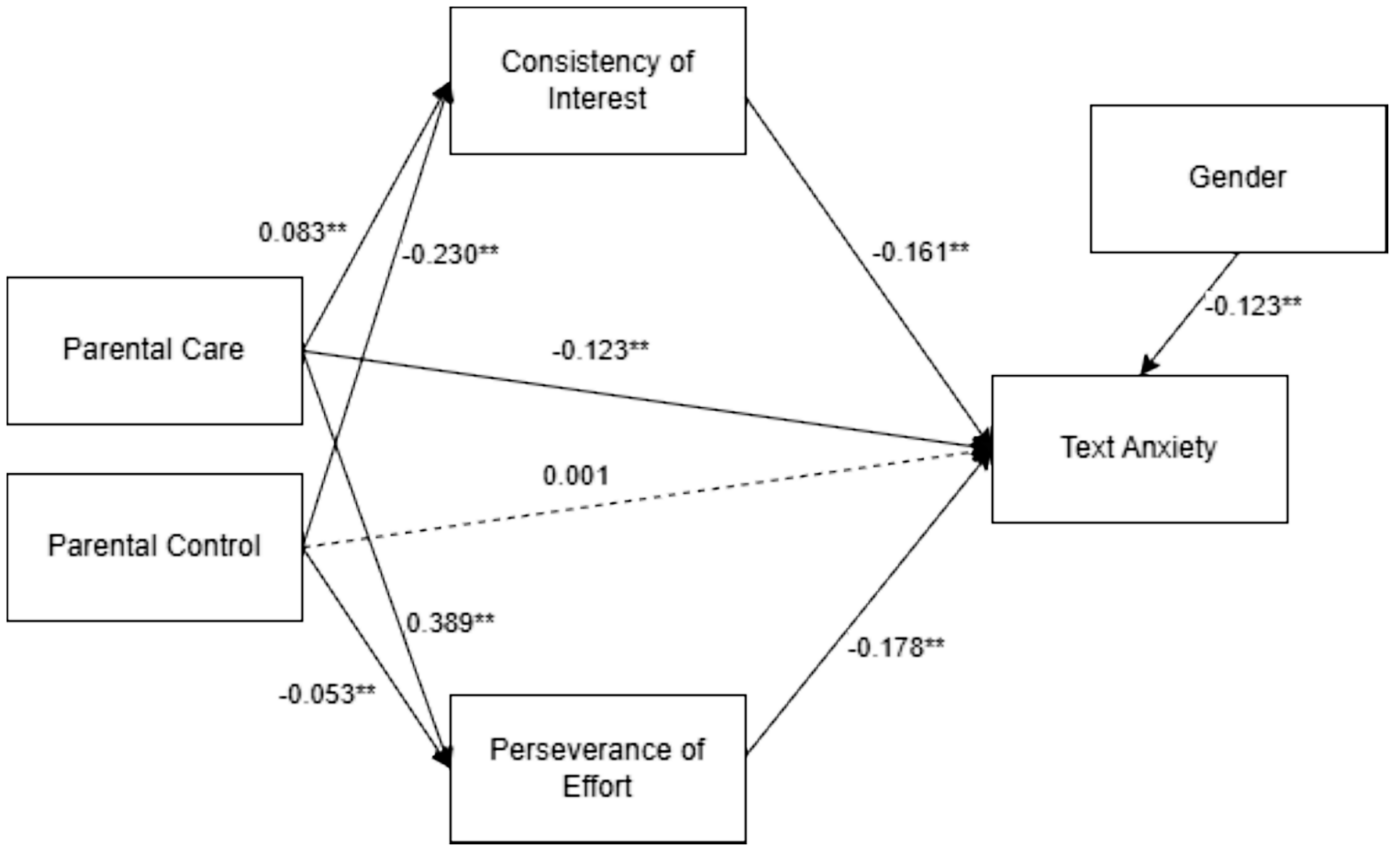
The model results with standardized path coefficient. *p < 0.05, **p < 0.01. Solid lines are for significant paths, and dotted lines are for insignificant paths.

As shown in Table 5, both perseverance of effort (*β* = −0.178, *p* < 0.001) and consistency of interest (*β* = −0.161, *p* < 0.001) were significant negative predictors of test anxiety, indicating that students with higher levels of grit reported lower anxiety. Parental care also showed a negative association with test anxiety (*β* = −0.123, *p* < 0.001), whereas the direct effect of parental control was minimal and non-significant (*β* = 0.0001, *p* = 0.985). Figure 1 illustrates the corresponding path coefficients.

**Table 5:** Direct path coefficients in the structural equation model (Estimator: ML).

Outcome	Predictor	Std. estimate	SE	*z*	*p*	95% CI (lower–upper)
Perseverance of effort	Parental Control	−0.053	0.006	−9.421	< 0 0.001	[−0.064, −0.042]
	Parental Care	0.389	0.005	77.209	< 0.001	[0.379, 0.399]
Consistency of interest	Parental Control	−0.230	0.006	−40.138	< 0.001	[−0.242, −0.219]
	Parental Care	0.083	0.006	13.967	< 0.001	[0.071, 0.095]
Test anxiety	Parental Control	0.0001	0.006	0.019	0.985	[−0.012, 0.012]
	Parental Care	−0.123	0.006	−19.664	< 0.001	[−0.135, −0.111]
	Perseverance of Effort	−0.178	0.006	−29.006	< 0.001	[−0.190, −0.166]
	Consistency of Interest	−0.161	0.006	−27.662	< 0.001	[−0.173, −0.150]
	Gender (Male = 1)	−0.155	0.006	−27.407	< 0.001	[−0.166, −0.144]

Regarding the predictors of grit, parental care had a strong positive effect on perseverance of effort (*β* = 0.389, *p* < 0.001) and a smaller but significant positive effect on consistency of interest (*β* = 0.083, *p* < 0.001). In contrast, parental control negatively predicted both perseverance (*β* = −0.053, *p* < 0.001) and consistency (*β* = −0.230, *p* < 0.001). Together, these results suggest that supportive parenting is associated with greater perseverance and sustained interest, whereas controlling parenting is linked to reductions in both dimensions of grit. These patterns ultimately help explain how parenting contributes to variations in adolescents’ test anxiety.

#### 3.3.2 Indirect

As shown in Table 6, both dimensions of grit significantly mediated the associations between parenting styles and test anxiety. The indirect effects of parental control on test anxiety were positive through both perseverance of effort (*β* = 0.009, *p* < 0.001) and consistency of interest (*β* = 0.037, *p* < 0.001), indicating that controlling parenting increases anxiety by diminishing students’ grit-related resources. In contrast, parental care showed significant negative indirect effects via perseverance (*β* = −0.069, *p* < 0.001) and consistency (*β* = −0.013, *p* < 0.001), suggesting that caring parenting reduces test anxiety by strengthening adolescents’ perseverance and sustained interest. These results underscore the mediating role of grit as a key psychological mechanism through which parenting styles exert their influence on students’ emotional outcomes.

**Table 6:** Indirect effects in the structural equation model (Estimator: ML).

Path	Std. estimate	SE	*z*	*p*	95% CI (lower–upper)
Parental control → Perseverance → Anxiety	0.009	0.001	8.952	< 0.001	[0.007, 0.012]
Parental care → Perseverance → Anxiety	−0.069	0.003	−26.937	< 0.001	[−0.074, −0.064]
Parental control → Consistency → Anxiety	0.037	0.002	22.589	< 0.001	[0.034, 0.040]
Parental care → Consistency → Anxiety	−0.013	0.001	−12.464	< 0.001	[−0.016, −0.011]

#### 3.3.3 Total effect

As summarized in Table 7, parental control demonstrated a small but significant positive total effect on test anxiety (*β* = 0.047, *p* < 0.001), indicating that higher levels of controlling parenting were associated with elevated anxiety. In contrast, parental care exerted a substantial negative total effect on test anxiety (*β* = −0.206, *p* < 0.001), suggesting that warm and supportive parenting is linked to reduced anxiety levels. These total effects reflect the combined influence of both direct and indirect pathways through the two grit dimensions, underscoring the broader role of parenting practices in shaping adolescents’ emotional responses.

**Table 7:** Total effects in the structural equation model (Estimator: ML).

Predictor → outcome	Std. estimate	SE	*z*	*p*	95% CI (lower–upper)
Parental control → Test anxiety	0.047	0.006	7.838	<0.001	[0.035, 0.059]
Parental control → Perseverance	−0.053	0.006	−9.421	<0.001	[−0.064, −0.042]
Parental control → Consistency	−0.230	0.006	−40.138	<0.001	[−0.242, −0.219]
Parental care → Test anxiety	−0.206	0.006	−35.543	<0.001	[−0.217, −0.194]
Parental care → Perseverance	0.389	0.005	77.209	<0.001	[0.379, 0.399]
Parental care → Consistency	0.083	0.006	13.967	<0.001	[0.071, 0.095]
Perseverance → Test anxiety	−0.178	0.006	−29.006	<0.001	[−0.190, −0.166]
Consistency → Test anxiety	−0.161	0.006	−27.662	<0.001	[−0.173, −0.150]
Gender → Test anxiety	−0.155	0.006	−27.407	<0.001	[−0.166, −0.144]

## 4 Discussion

The present study examined the relationship between parenting styles and adolescent test anxiety, with a focus on the mediating role of academic grit. Methodologically, the research combined partial correlation network analysis and structural equation modeling (SEM). Across analytic approaches, academic grit-particularly perseverance of effort-emerged as a central construct in the relational network linking family socialization practices and students’ emotional responses in evaluative contexts. These findings extend current perspectives on academic motivation and anxiety by positioning academic grit not solely as an individual trait, but as a psychological process associated with patterns of emotional adjustment within family contexts.

### 4.1 Mediating mechanism of academic grit

Across the two analytic approaches, academic grit demonstrated robust negative associations with test anxiety, although the two methods address different structural aspects of the system. In the network analysis, perseverance of effort exhibited the highest structural centrality, indicating that it occupied a prominent position within the pattern of conditional associations linking parenting variables and outcomes. This structural prominence reflects its integrative role within the associative configuration of the network.

Within the SEM framework, the relations among parenting styles, the two dimensions of academic grit, and test anxiety were examined using a theoretically specified directional model. The results indicated that parental care was positively associated with perseverance of effort and consistency of interest, both of which were negatively associated with test anxiety. Parental control showed negative associations with the two grit dimensions and positive indirect associations with anxiety. These indirect associations were statistically significant based on bootstrapped confidence intervals.

These findings are consistent with prior literature indicating that supportive parenting is associated with higher levels of autonomy, motivation, and self-regulatory capacities (Ryan and Deci, 2000), whereas elevated psychological control tends to be linked with lower intrinsic motivation and greater emotional strain (Grolnick and Pomerantz, 2009). The present results extend this line of work by situating academic grit within the relational pattern connecting parenting styles and test anxiety-a long-term, effort-focused characteristic that appears particularly salient in high-stakes learning environments.

Importantly, perseverance of effort demonstrated larger indirect associations with test anxiety than consistency of interest within the SEM model. In East Asian schooling contexts, where diligence and sustained academic engagement are culturally emphasized, perseverance may carry greater cultural salience and be more closely aligned with prevailing educational values (Datu et al., 2017). This pattern points to potential cultural variation in the relative functional relevance of academic grit’s subcomponents and underscores the importance of interpreting grit within culturally grounded rather than universalized frameworks.

### 4.2 Cultural and psychological implications

Interpreting the findings within an East Asian cultural context provides important insight into the observed mechanisms. In societies characterized by high parental involvement and strong academic expectations, children often internalize achievement-related norms as moral obligations (Li, 2003). Within such environments, academic striving is not merely an individual preference but a socially endorsed duty. Supportive parenting may therefore be associated with more adaptive internalization of these expectations, allowing adolescents to relate externally structured goals to personally endorsed commitments. In contrast, controlling parenting may be linked to higher performance pressure, greater fear of failure, and elevated anxiety when academic standards are perceived as externally imposed (Pomerantz and Wang, 2009).

From the perspective of Self-Determination Theory (Ryan and Deci, 2000), these patterns may reflect differences in motivational internalization. Parental care can provide autonomy support and emotional security, fostering identified or integrated forms of regulation that sustain effort with reduced emotional cost. Conversely, controlling practices may promote externally regulated motivation, in which academic engagement is driven by obligation or avoidance of negative evaluation. In this sense, academic grit may function as a behavioral manifestation of internalized motivation, linking parenting environments to emotional outcomes.

A complementary social-cognitive explanation further clarifies the process. Supportive parental feedback may be associated with higher adolescents’ self-efficacy and perceived competence, which in turn tends to relate to greater persistence and lower anticipatory anxiety. By contrast, excessive control may undermine confidence and heighten self-doubt, increasing vulnerability to evaluative stress. Under this framework, academic grit reflects a motivational-cognitive resource embedded within students’ belief systems rather than solely a dispositional trait.

These interpretations can also be viewed through a psychodynamic lens, which emphasizes how early relational experiences shape internalized authority and self-evaluative processes. Warm, responsive parenting may foster a cohesive sense of self capable of regulating achievement pressures (Zeanah, 1990), whereas controlling parenting may intensify internal tensions between self-expectations and perceived standards, manifesting as anxiety in evaluative situations. Rather than privileging a single explanatory model, the findings likely reflect the intersection of motivational internalization, cognitive self-beliefs, and relational dynamics.

Culturally, the results also contribute to ongoing debates regarding the meaning of academic grit across contexts. Whereas Western conceptualizations emphasize passion-driven goal pursuit, academic grit among Chinese adolescents may reflect a form of moralized persistence shaped by family and societal expectations (Heine et al., 1999). In this setting, perseverance may be rooted less in individual preference and more in a culturally reinforced ethic of effort. This interpretation aligns with cross-cultural findings suggesting that perseverance of effort carries stronger adaptive value than consistency of interest in collectivist contexts (Lam and Zhou, 2022). The present results, in which perseverance played a more prominent mediating role than consistency, are consistent with this culturally embedded understanding of academic grit.

## 5 Conclusion and practical implications

The present study deepens our understanding of how parenting shapes adolescents’ emotional experiences by identifying academic grit as a central pathway through which family practices influence test anxiety. Using a large and representative sample of Chinese middle school students, the combined application of network analysis and structural equation modeling showed consistent patterns: supportive parenting was associated with higher academic grit and lower anxiety, whereas controlling parenting was linked to lower academic grit and higher anxiety. The two facets of academic grit-perseverance of effort and consistency of interest—played different mediating roles, with perseverance emerging as the more protective factor. These findings highlight the multidimensional nature of academic grit and its important function in helping adolescents manage stress in highly demanding academic contexts.

Building on these insights, the results point to the value of involving families in efforts to reduce test anxiety. Parents should understand that students’ emotional distress does not stem solely from school demands or examination pressures. Family interactions exert strong psychological effects by shaping adolescents’ motivation, coping styles, and self-regulation abilities. Raising parental awareness and encouraging healthier communication and expectations are therefore essential components of effective prevention and intervention programs.

Several practical strategies may help achieve such cultural adaptation. First, parent-focused interventions could emphasize autonomy-supportive communication while maintaining culturally valued academic standards. Rather than reducing expectations, programs may guide parents in shifting from outcome-oriented pressure (“achieve high scores”) to process-oriented encouragement (“recognize effort and strategy use”). Workshops or structured family dialogs could train parents to provide informational feedback, validate emotional experiences during exams, and differentiate between high expectations and psychological control. Such adjustments align with the finding that parental care is associated with higher perseverance without a corresponding increase in anxiety.

Second, school-based programs may integrate culturally resonant narratives of effort with emotional regulation skills. For example, perseverance can be framed not only as endurance under pressure but as strategic, self-directed commitment. Classroom interventions might combine goal-setting exercises with reflective discussions on managing evaluative stress, helping students reinterpret academic effort as self-endorsed growth rather than externally imposed obligation. Embedding stress-management techniques within discussions of diligence and responsibility may enhance acceptability in collectivist settings.

Third, collaborative family-school initiatives could establish consistent messaging around adaptive persistence. Educators and counselors may provide parents with concrete guidelines on balancing involvement and autonomy, such as structured homework routines that allow student choice, or performance reviews that include both achievement metrics and emotional well-being indicators. Such systemic alignment may prevent controlling practices from inadvertently undermining motivational resources.

More broadly, cultural adaptation requires recognizing that perseverance in East Asian contexts may already be high but emotionally costly. Interventions should therefore focus less on amplifying effort and more on reshaping the psychological meaning of effort-from obligation-driven endurance to internally regulated commitment. By doing so, culturally grounded programs can preserve valued norms of diligence while reducing their unintended emotional burden.

### 5.1 Limitation and future direction

Despite the study’s large sample size and careful research design, several limitations should be noted.

First, the data were collected through cross-sectional self-report questionnaires, which limits the ability to determine how parenting styles, academic grit, and test anxiety influence one another over time. Future research could use longitudinal or experimental designs to clarify the order in which these factors develop, identify their causal directions, and explore whether relationships between family dynamics and adolescents’ emotional adjustment may be bidirectional.

Second, although the sample drawn from Haidian District in Beijing is highly representative within the Chinese educational setting, the findings may not fully apply to adolescents from different socioeconomic or cultural contexts. Future studies should broaden sampling to include rural areas, multiple provinces, and cross-cultural comparisons in both East Asian and Western settings. Such work would help determine whether the mediating role of academic grit is broadly shared across cultures or shaped by values rooted in Confucian traditions.

Third, the study did not collect detailed socioeconomic indicators such as parental education level, occupational status, or household income. Because the survey was conducted as part of a large-scale district-level assessment initiative, sociodemographic variables were not included in order to reduce participant burden and ensure high response compliance. Nevertheless, socioeconomic status (SES) may play a meaningful role in shaping parenting practices, adolescents’ motivational development, and emotional adjustment. The absence of SES measures limits the ability to examine potential moderating or confounding effects associated with family background. Future research should incorporate multidimensional SES indicators to assess whether the observed pathways differ across socioeconomic strata.

Fourth, this study relied largely on students’ self-reported perceptions of parenting and emotional experiences, which may be affected by social desirability or memory bias. Including information from multiple sources—such as parents and teachers—or using more objective behavioral indicators of academic grit and test anxiety would strengthen the reliability of the results.

Finally, the current model examined academic grit as the only mediating factor, yet other personal traits, including self-efficacy, emotion regulation, and academic resilience, may also influence how parenting relates to adolescents’ anxiety. Future research could incorporate these additional factors into broader models to better reflect the complex interactions among family influences, motivation, and emotional well-being.

In sum, although the present study offers meaningful insights into how parenting relates to emotional outcomes among East Asian adolescents, ongoing research using longitudinal, cross-cultural, and multi-method approaches will be crucial for refining theoretical understanding and guiding more culturally responsive interventions.

## Data Availability

The raw data supporting the conclusions of this article will be made available by the authors, without undue reservation.
